# Examination of Transition Shock and Colleague Violence Among Newly Graduated Nurses: A Cross-Sectional Study

**DOI:** 10.1155/2024/5486048

**Published:** 2024-10-17

**Authors:** Soner Berşe, Ali Ağar, Ezgi Di̇rgar, Betül Tosun

**Affiliations:** ^1^Department of Nursing, Faculty of Health Sciences, Gaziantep University, Gaziantep, Türkiye; ^2^Department of Elderly Care, Şavşat Vocational School, Artvin Çoruh University, Artvin, Türkiye; ^3^Department of Midwifery, Faculty of Health Sciences, Gaziantep University, Gaziantep, Türkiye; ^4^Faculty of Nursing, Hacettepe University, Ankara, Türkiye

**Keywords:** early career nurses, nurse education, professional development, student nurses, transition shock, transition to practice, workplace violence

## Abstract

**Background:** The adaptation process for new nursing graduates is challenging, with transition shock and colleague violence impacting both individuals and institutions.

**Objective:** This study investigates transition shock and colleague violence among newly graduated nurses in Turkey during their adaptation process.

**Methods:** The study involved 235 newly graduated nurses from a state university in Turkey with at least six months of clinical experience. Data were collected using the Nursing Transition Shock Scale and the Exposure to Colleague Violence Scale.

**Results:** Among the participants, 27.23% experienced colleague violence and 56.17% witnessed it. The mean score on the Nursing Transition Shock Scale was 53.62 ± 15.39. Female nurses and younger age groups faced more challenges.

**Conclusion:** Supportive work environments, mentorship programs, and collaborative teamwork are crucial for newly graduated nurses. Updating nursing education programs to prepare students for these challenges is essential. This study underscores the need for targeted interventions.

## 1. Introduction

As fundamental healthcare professionals, nurses constitute over half of the global healthcare workforce, representing a community of approximately 28 million individuals [1]. The global shortage in nursing is alarming in many countries, with a significant demand for new graduates to fill the workforce gap by 2025 due to retirements and resignations [2]. The concept of transition shock was first described by Kaihlanen, Hietapakka, and Heponiemi to define the situation where newly graduated nurses experience anxiety, insecurity, insufficiency, and imbalance. If the increased responsibilities and stress following graduation are overlooked, this transition shock can complicate adaptation to working life [3]. A study by Labrague and Santos detailed the effects of transition shock on job and patient outcomes among new graduate nurses, showing a direct relation with job satisfaction, stress, burnout, and impacting patient care quality [4]. Moreover, research by Zhao et al. observed the adverse effects of challenges faced by nursing interns during clinical internships on their career decisions and attitudes toward patient safety, exploring the relationship between professional identity and care climate for new graduate nurses [5]. Hence, the difficulties and colleague violence experienced by newly graduated nurses during their transition to working life are closely linked to personal experiences, the quality of healthcare services, and patient safety [6].

Globally, the healthcare services, particularly the nursing workforce, face historic recruitment and retention challenges [7]. The transition to working life is a tumultuous time for new graduate nurses, as they are vulnerable newcomers needing understanding and support from more experienced colleagues. This period warrants investigation into whether this group is ready to work and how best to integrate these new nurses into clinical practice, considering the issues surrounding their entry into the healthcare system workforce [6, 8].

Workplace violence can manifest as horizontal violence among peers or vertical violence from managers and superiors [9]. Violent behaviors can negatively impact employees, leading to decreased quality of patient care, intentions to leave the nursing profession, further organizational inefficiency, and adverse patient experiences, thereby perpetuating a cycle of violence [10]. Newly graduated nurses lacking support from colleagues and managers may be subjected to bullying, violence, job dissatisfaction, and stress, affecting their commitment to the profession from the early years of employment [2]. In addition, the undesirable outcomes of new graduates feeling emotionally exhausted, unable to meet job demands, providing safe care, and feeling unsupported could increase the likelihood of making errors [11].

Current literature suggests that the challenges faced by newly graduated nurses should be addressed not only at the individual level but also at institutional and systemic levels [1, 2].

This study makes a significant and novel contribution to the literature by being one of the first to integrate the concepts of transition shock and colleague violence among newly graduated nurses. Unlike previous research, which has typically examined these two phenomena in isolation, this study explores their interaction and how they collectively impact newly graduated nurses [1, 2], providing a fresh perspective on these critical issues. By focusing on the context of Turkey—a region underrepresented in global literature—the study also offers unique insights that can serve as a valuable reference for similar settings in other countries. Furthermore, the use of comprehensive data analyses and advanced statistical methods enables a more nuanced and in-depth examination of the relationship between transition shock and colleague violence, potentially guiding future research and interventions in this area. Therefore, this study aims to make significant contributions toward understanding the experiences of transition shock and colleague violence among newly graduated nurses.

## 2. Materials and Methods

### 2.1. Design

This descriptive cross-sectional study employed to examine the experiences of transition shock and colleague violence among newly graduated nurses in Turkey. The research was conducted between June and September 2023.

### 2.2. Study Sample

The study was conducted with newly graduated nurses who had graduated from the Nursing Department of a state university in Turkey within the last 2 years and had at least 6 months of clinical experience.

#### 2.2.1. Inclusion Criteria

• Newly graduated nurses within the last 2 years.
• Completion of education in the Nursing Department.
• A minimum of 6 months of clinical experience.
• Willingness to voluntarily participate in the study.

#### 2.2.2. Exclusion Criteria

• Nurses with less than 6 months of clinical experience.
• Nurses who were not currently employed in a clinical setting.

The sample size and statistical power were calculated using *G*^∗^ Power analysis. Also, a priori power analysis was conducted based on a one-tailed test, a medium effect size (*d* = 1.37), a 5% alpha error level, and 95% power (1-*β*) parameters [12]. According to these calculations, 132 participants were sufficient for the study.

### 2.3. Data Collection Method

Our data collection process was conducted through a platform established for postgraduation follow-up purposes. These groups encompass 450 students who graduated from the Nursing Department of a state university in Turkey in the last two years. These students actively participate in groups established to share their clinical experiences, support their professional development, and provide academic counseling.

An informed invitation message about the purpose and importance of our research was sent to all members of these groups. The message highlighted the voluntary nature of participation, the anonymity of the data collection, and the fact that the results would be used solely for academic purposes. It also emphasized that participation in the study would contribute to the knowledge base in our field.

The survey link was distributed via a social media platform directly to the members of the graduation follow-up groups. This method aimed to reach each of the 450 newly graduated nurses who graduated in the last two years and had at least six months of clinical experience. In total, 235 graduate nurses responded to our survey by filling it out, indicating a response rate of 52.2%.

Throughout the data collection process, the anonymity and privacy of the participants were maintained at the highest level. Data collected via Google Forms did not include personal identifying information, thereby fully protecting the participants' privacy. Ethical approval for our research was obtained, and participants were presented with an informed consent form before completing the survey. This form clearly outlined the study's purpose, the participants' rights, and how the collected data would be used.

### 2.4. Data Collection Instruments

#### 2.4.1. Personal Information Form

The Personal Information Form consists of eight items designed to understand participants' demographic and professional profiles. The survey includes questions about demographic information such as age, gender, reasons for choosing the profession, and professional experiences such as thoughts of leaving the profession and experiences of colleague violence [8–10, 13, 14].

#### 2.4.2. Nursing Transition Shock Scale (NTSS)

The NTSS, developed by Tarhan and Yıldırım, is an 18-item scale used to assess the adaptation process of newly graduated nurses in various dimensions during their transition to working life [15]. Each item is rated on a five-point Likert scale, covering four subdimensions: “Perception of Inadequacy in Interpersonal Relationships (PIIR),” “Perception of Inadequacy in Professional Decisions and Practices (PIPD),” “Perception of Inadequacy in Social Life (PISL),” and “Perception of Inadequacy in Roles and Responsibilities (PIRR).” The original scale had a total Cronbach's alpha coefficient of 0.92, while this study found it to maintain a similar reliability level, with a total Cronbach's alpha of 0.95. The coefficient for the subdimension of perceived inadequacy in interpersonal relationships was 0.88 in the original scale and 0.92 in this study; for the subdimension of perceived inadequacy in professional decisions and practices, it was 0.84 originally and 0.83 in this study. Similarly, the coefficient for the subdimension of perceived inadequacy in social life was 0.80 originally and 0.87 in this study. Finally, the subdimension of perceived inadequacy in roles and responsibilities was 0.80 originally and 0.84 in this study.

#### 2.4.3. Exposure to Colleague Violence Scale for Nursing Students (ECVSN)

The ECVSN, developed by Yılmaz, Ata, and Uyumaz, is a 22-item, 5-point Likert scale designed to assess nursing students' exposure to colleague violence in the clinical setting [16]. This scale measures Verbal/Psychological Violence dimension (VVD) and the effects of violence on physical and mental health. The original study reported a total Cronbach's alpha coefficient of 0.944, with subdimensions of “exposure to VVD” at 0.931 and “effects of violence on physical and mental health” at 0.886. In this study, the overall Cronbach's alpha for the Exposure to Colleague Violence Scale (CVES) was 0.97, with 0.94 for the “VVD” and 0.96 for the “Effects of Violence on Physical and Mental Health Dimension.”

### 2.5. Ethical Approval

This research has been approved by the Artvin University Scientific Research and Publication Ethics Committee (11.01.2023, E.77360). The data were collected with the study permit number E-87841438-900-346469 dated 23.06.2023 from Gaziantep University. The study was designed and conducted using the principles of the Declaration of Helsinki. All participants involved in the study were informed about the research and assured that they could withdraw from the study at any time. Furthermore, voluntary consent forms obtained from participants ensured their rights were protected, and their participation in the research was based entirely on voluntary consent.

### 2.6. Statistical Analysis

Data analysis was performed using SPSS and Amos software. Descriptive statistics for the participants' demographic characteristics were calculated, and relationships between scale scores were tested using Pearson correlation analysis. Independent samples *t*-tests and one-way analysis of variance (ANOVA) were applied to determine differences between groups. Path analysis was employed to assess the structural relationships between the perceptions of inadequacy in various domains (interpersonal relationships, professional decisions and practices, social life, and roles and responsibilities) and exposure to colleague violence. The analysis was conducted by calculating standardized and unstandardized estimates, along with standard errors (SE), critical ratios (CR), and *p* values for each path in the model. The significance level for all statistical tests was set at 0.05.

## 3. Results

The mean age of the participants was 22.58 ± 3.5 years, and the mean of working year was 1.52 ± 2.81 years. Of the nurses, 81.70% were female and 61.70% chose their profession willingly. 30.64% of the participants considered leaving the profession, 27.23% were exposed to colleague violence, and 56.17% witnessed colleague violence (Table 1). The mean score obtained by newly graduated nurses from the CVES was 59.26 ± 20.7 (min = 22-max = 110). The mean score for the Verbal/Psychological Violence subdimension of the scale was 28.3 ± 9.92, and for the Effects of Violence on Physical and Mental Health subdimension, it was 30.96 ± 11.63. The mean score from the NTSS was 53.62 ± 15.39, with the subdimension scores for PIIR at 19.97 ± 6.55, PIPD at 11.95 ± 3.61, PISL at 12.72 ± 4.22, and PIRR at 9.00 ± 3.01 (Table 2). When examining the mean scores of the CVES/Subdimension and the NTSS/Subdimension according to the gender of the participants, it was found that female nurses scored statistically significantly higher on both the CVES/Subdimensions (*t* = 2.939, *t* = 2.527, and *t* = 3.072, respectively, *p* < 0.05) and the NTSS/Subdimensions compared to male nurses (*t* = 2.262, *t* = 2.011, *t* = 2.063, *t* = 1.719, and *t* = 2.187, respectively, *p* < 0.05). A statistically significant difference was found between the age groups of newly graduated nurses and the total mean score of the CVES/Subdimension. Further analysis showed that nurses in the age range of 21–23 years had statistically significantly higher scores compared to those aged 18–20 and over 24 years (*F* = 4.042, *F* = 3.109, and *F* = 4.806, respectively, *p* < 0.05).

A statistically significant difference was detected between the condition of considering leaving the profession and the mean total scores of the CVES/Subdimension and the NTSS/Subdimension. Accordingly, newly graduated nurses who considered leaving the profession had higher mean scores than those who did not (*t* = 6.269, *t* = 5.402, and *t* = 6.499, respectively, *p* < 0.05). Significant differences were found between the conditions of experiencing and witnessing colleague violence and the scores of the CVES/Subdimension, NTSS, and its subdimensions of PIIR, PISL, and PIRR. Accordingly, new graduates who were exposed to or witnessed colleague violence had higher mean scores compared to those who did not experience violence (*t* = 7.454, *t* = 6.913, and *t* = 7.255, respectively,*p* < 0.05) (Table 3).

**Figure 1:**
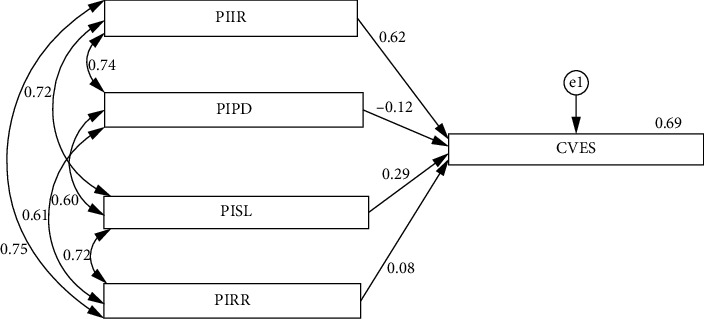
Path analysis of the relationship between nurses' Nursing Transition Shock Scale subdimensions and Exposure to Colleague Violence Scale. Abbreviations: CVES = Exposure to Colleague Violence Scale, PIIR = Perception of Inadequacy in Interpersonal Relationships dimension, PIPD = Perception of Inadequacy in Professional Decisions and Practices dimension, PIRR = Perception of Inadequacy in Roles and Responsibilities dimension, PISL = Perception of Inadequacy in Social Life dimension.

It was observed that the PIIR, PIPD, and PISL significantly influenced nurses' CVES (*β* = 0.622, *β* = −0.12, and *β* = 0.287, respectively, *p* < 0.05). At the same time, the perception of inadequate roles and responsibilities did not show a significant effect (Table 4). The subdimensions of the NTSS explained 69% of the variance (*R*^2^ = 0.69) (Figure 1).

## 4. Discussion

The initiation period into the workforce for newly graduated nurses brings numerous challenges [4, 5, 14]. The key findings of the study highlight the significant impact of demographic characteristics on newly graduated nurses' experiences with colleague violence and transition shock. Factors such as gender, age, and the contemplation of leaving the profession play a crucial role in these experiences, underscoring the prevalence and profound effects of these challenges within the nursing workforce. Specifically, the study found that female nurses experience these difficulties more than their male counterparts, with younger age groups being particularly vulnerable. In addition, nurses considering leaving the profession scored higher on both the CVES and the NTSS, suggesting that colleague violence and transition shock are significant factors influencing thoughts of leaving the profession. This is consistent with studies in the literature, which emphasize that female nurses are more exposed to colleague violence compared to male nurses, a situation often linked to societal expectations and social responsibilities outside of work [14, 16].

Contextualizing these findings within the broader literature, Cai emphasizes the influence of demographic variables such as gender and educational background on work adaptability, suggesting that nurse managers must be aware of the diverse backgrounds from which newly graduated nurses enter the profession [12]. This aligns with our study's findings, as demographic factors are shown to predict experiences of violence and transition shock. Moreover, the scoping review by Alshawush, Hallett, and Bradbury-Jones supports the notion that while transition programs (TPs) can assist in easing the transition for newly graduated nurses, they are not entirely effective in preventing issues like workplace violence and stress, further reinforcing the necessity of considering demographic factors when designing these programs [2]. In addition, research by Dertli and Alttura and Yao et al. illustrates how transition shock is influenced by various factors, including the nurse's background and psychological wellbeing, with Yao et al. noting that psychological transition shock is particularly pronounced, suggesting the critical need for tailored support mechanisms to address these challenges, especially considering their long-term impact [17, 18].

The impact of demographic characteristics of newly graduated nurses on the results of the CVES and NTSS, related to factors such as gender, age, and consideration of leaving the profession, unveils the prevalence and effects of these challenges among nurses. In this study, it was found that female nurses experience these difficulties more than their male counterparts, and this situation is more pronounced in younger age groups. Moreover, nurses considering leaving the profession scored higher than those not considering leaving, suggesting that colleague violence and transition shock might influence thoughts of leaving the profession. Studies in the literature emphasized that female nurses are more exposed to colleague violence compared to male nurses, and this can be associated with societal expectations and social responsibilities outside of work [14, 16].

In addition, the impact of violence on nurses' physical and mental health can adversely affect their sense of professional integrity and self-perception [5]. Female senior nursing students experienced more difficulties in the transition to work than males, and a desire to be a nurse and loved for the profession positively affected transition shock [5]. Transition shock experiences of senior nursing students were related to excessive workload, limitations of professional knowledge, and conflicts between theoretical and practical applications [13]. Nurses in private hospitals had higher perceptions of transition shock than those in public hospitals, indicating the significant effect of the working environment on nurses' perceptions of transition shock [4]. In light of these findings, the importance of mentorship programs, workload management, and psychosocial support to enhance the overall quality of nursing practice and ensure a successful transition into work life for newly graduated nurses is emphasized. These strategies can support nurses in coping with challenges such as colleague violence and transition shock, reducing thoughts of leaving the profession and fostering a healthier work environment in the healthcare services.

In this study, the effects of the subdimensions of the NTSS on exposure to colleague violence were revealed through path analysis, presenting significant findings in the nursing field. Notably, the positive and robust impact of the PIIR on colleague violence was consistent with existing literature. These results aligned with previous studies that showed the significant role of interactions among nurses and the quality of the work environment on tendencies toward violence [2, 19, 20].

The finding that the PIPD is a mitigating factor for colleague violence highlights the importance of enhancing nurses' professional competencies and decision-making processes. This contributes to the existing literature by suggesting that developing decision-making skills can positively affect the work environment [21, 22].

The positive effects of PISL and roles and responsibilities on violence indicate that the balance between work and personal life can impact professional satisfaction and perceptions of violence in the workplace [23]. These findings were consistent with previous studies supporting the idea that social support systems and clarity in role definitions at the workplace can reduce perceptions of violence [24]. However, the nonsignificant effect of the PIRR in our study suggests that its impact on colleague violence might be less pronounced compared to other dimensions. In light of these findings, it is concluded that nursing education programs and work environments should focus on developing interpersonal communication skills and professional competencies. Designing nursing education and workplace policies to develop skills to cope with these challenges may contribute to preventing colleague violence [25].

### 4.1. Limitations

The results of this study are limited to the responses of newly graduated nurses who have graduated from the one state university and started working and cannot be generalized to the entire population. In addition, it should be noted that participants might have provided biased responses, and thus caution is advised in extrapolating these findings.

## 5. Conclusion

This study makes a significant contribution to understanding the complex challenges faced by newly graduated nurses, specifically the prevalence of colleague violence and transition shock, and their impact on job satisfaction and retention. Unlike previous research, this study offers practical applications and policy recommendations for addressing these issues at both individual and systemic levels. It proposes strategies such as supportive work environments, mentorship programs, and enhanced communication skills training, filling a critical gap in the literature and laying the groundwork for future research on effective interventions.

The study highlights that new graduate nurses face significant challenges, with colleague violence and transition shock notably affecting job satisfaction and retention. Healthcare institutions and nursing education programs should develop supportive strategies, including creating positive work environments, implementing mentorship programs, fostering teamwork, and enhancing communication skills. These measures are essential for managing the transition effectively and preparing students to handle such challenges.

In conclusion, collaboration between nurse educators and healthcare providers is crucial for developing policies and programs that ease the transition and ensure the profession's sustainability and healthcare quality. Future research should explore transition shock further and consider experimental studies focused on reducing these challenges.

### 5.1. Implications for Nursing Management

Nursing management plays a crucial role in addressing the challenges faced by newly graduated nurses, particularly in mitigating colleague violence and transition shock. To create a supportive work environment, managers should foster a culture of open communication, ensuring that new nurses feel safe and supported. Implementing effective mentorship programs can significantly aid in easing the transition process by providing guidance and reducing the stress associated with adapting to the new role. Training in communication skills is essential to help nurses manage conflicts and build healthier interpersonal relationships, which can decrease instances of colleague violence. In addition, managing workload and promoting work–life balance are critical to prevent burnout and dissatisfaction. Nursing management should also review and update policies and procedures to address issues related to colleague violence and transition shock. Establishing psychosocial support systems, such as counseling and support groups, can further assist nurses in navigating the challenges of their new roles. Lastly, collecting and acting on feedback from nurses can help in continuously improving management practices to better support newly graduated nurses.

## Figures and Tables

**Table 1:** Distribution of nurses' descriptive characteristics (*n*: 235).

Characteristics	*n*	%
Age (years): 22.58 ± 3.5 (18–48)		
18–20	40	17.02
21–23	157	66.81
24–+	38	16.17
Years of work: 1.52 ± 2.81 (0–27)		
Gender		
Female	192	81.70
Male	43	18.30
Choosing the profession willingly		
I chose it myself	145	61.70
Chosen due to my family's wish	77	32.77
Chosen with my counselor teacher	13	5.53
Considering leaving the profession?		
Yes	72	30.64
No	163	69.36
Exposed to colleague violence?		
Yes	64	27.23
No	171	72.77
Witnessed colleague violence?		
Yes	132	56.17
No	103	43.83
Unit worked in		
Ward	122	51.91
Intensive care	20	8.51
Emergency	20	8.51
Outpatient clinic	7	2.98
Operating room	4	1.70
Other	62	26.38

**Table 2:** Average scores of nurses on the Exposure to Colleague Violence Scale/Subdimensions and Nursing Transition Shock Scale/Subdimensions.

Exposure to Colleague Violence Scale and its Subdimensions	Cronbach's alpha	Mean ± SD	Min–max
Exposure to Colleague Violence Scale	0.97	59.26 ± 20.7	22–110
Verbal/psychological violence dimension	0.94	28.3 ± 9.92	11–55
Effects of violence on physical and mental health dimension	0.96	30.96 ± 11.63	11–55

*Nursing Transition Shock Scale and its subdimensions*			
Nursing Transition Shock Scale	0.95	53.62 ± 15.39	18–90
Perception of inadequacy in interpersonal relationships dimension	0.92	19.97 ± 6.55	7–35
Perception of inadequacy in professional decisions and practices dimension	0.83	11.95 ± 3.61	4–20
Perception of inadequacy in social life dimension	0.87	12.72 ± 4.22	4–20
Perception of inadequacy in roles and responsibilities dimension	0.84	9.00 ± 3.01	3–15

**Table 3:** Comparison of nurses' descriptive characteristics with scores on the Exposure to Colleague Violence Scale/Subdimensions and Nursing Transition Shock Scale/Subdimensions.

Characteristics	CVES	VVD	EVPM	NTSS	PIIR	PIPD	PIRR	PISL
*Gender*								
Female	61.1 ± 20.91	29.07 ± 10	32.04 ± 11.82	54.69 ± 15.82	20.37 ± 6.83	12.18 ± 3.67	12.95 ± 4.19	9.2 ± 3.01
Male	51 ± 17.71	24.89 ± 8.89	26.12 ± 9.45	48.87 ± 12.39	18.17 ± 4.81	10.94 ± 3.16	11.68 ± 4.27	8.1 ± 2.89
*t*/*p*	**2.939/0.004**⁣^∗^	**2.527/0.012**⁣^∗^	**3.072/0.002**⁣^∗^	**2.262/0.025**⁣^∗^	**2.011/0.045**⁣^∗^	**2.063/0.04**⁣^∗^	1.791/0.075	**2.187/0.03**⁣^∗^

*Age*								
18–20^(a)^	51.43 ± 20.36	24.78 ± 9.68	26.65 ± 11.37	49.8 ± 13.48	18.25 ± 5.33	11.93 ± 3.2	11.28 ± 4.49	8.35 ± 2.61
21–23^(b)^	61.58 ± 20.67	29.08 ± 10.05	32.51 ± 11.46	55.92 ± 15.31	20.95 ± 6.61	12.34 ± 3.54	13.32 ± 4.05	9.32 ± 3.06
24–+^(c)^	57.87 ± 19.5	28.82 ± 9.01	29.06 ± 11.45	48.14 ± 15.79	17.72 ± 6.67	10.37 ± 3.95	11.72 ± 4.17	8.35 ± 3.04
*F/P*	**4.042/0.019**⁣^∗^	**3.109/0.047**⁣^∗^	**4.806/0.01**⁣^∗^	**5.612/0.005**⁣^∗^	**5.612/0.005**⁣^∗^	**4.704/0.01**⁣^∗^	**5.199/0.007**⁣^∗^	2.734/0.068
Difference	*b* > *a*	*b* > *a*	*b* > *a*	*b* > *c*	*b* > *c*	*b* > *c*	*b* > *a*,c	—

*Considering leaving profession*								
Yes	71.06 ± 18.99	33.27 ± 9.16	37.8 ± 10.7	62.14 ± 12.52	23.45 ± 6.08	13.1 ± 3.13	15.12 ± 3.24	10.49 ± 2.53
No	54.04 ± 19.27	26.11 ± 9.46	27.94 ± 10.74	49.86 ± 15.07	18.43 ± 6.17	11.45 ± 3.7	11.66 ± 4.18	8.34 ± 2.97
*t*/*p*	**6.269/0.001**⁣^∗^	**5.402/0.001**⁣^∗^	**6.499/0.001**⁣^∗^	**6.057/0.001**⁣^∗^	**5.775/0.001**⁣^∗^	**3.311/0.001**⁣^∗^	**6.255/0.001**⁣^∗^	**5.361/0.001**⁣^∗^

*Exposed to colleague violence*								
Yes	74.07 ± 19.29	34.97 ± 10.08	39.1 ± 10.18	61.72 ± 13.93	23.47 ± 6.32	12.58 ± 3.68	15.22 ± 3.71	10.46 ± 2.78
No	53.71 ± 18.4	25.81 ± 8.65	27.91 ± 10.65	50.59 ± 14.84	18.66 ± 6.16	11.72 ± 3.57	11.78 ± 4.02	8.45 ± 2.92
t/*p*	**7.454/0.001**⁣^∗^	**6.913/0.001**⁣^∗^	**7.255/0.001**⁣^∗^	**5.206/0.001**⁣^∗^	**5.3/0.001**⁣^∗^	1.642/0.102	**5.978/0.001**⁣^∗^	**4.77/0.001**⁣^∗^

*Witnessed colleague violence*								
Yes	66.35 ± 21.32	31.72 ± 10.09	34.64 ± 12.14	57.79 ± 15.74	21.72 ± 6.95	12.57 ± 3.67	13.91 ± 4.16	9.6 ± 3.04
No	50.16 ± 15.82	23.93 ± 7.78	26.24 ± 9	48.29 ± 13.21	17.73 ± 5.24	11.16 ± 3.39	11.18 ± 3.79	8.23 ± 2.8
*t/p*	**6.447/0.001**⁣^∗^	**6.479/0.001**⁣^∗^	**5.879/0.001**⁣^∗^	**4.923/0.001**⁣^∗^	**4846/0.001**⁣^∗^	**3.031/0.003**⁣^∗^	**5.2/0.001**⁣^∗^	**3.547/0.001**⁣^∗^

*Note:F* = ANOVA, *T* = independent sample t-test.

Abbreviations: CVES = Exposure to Colleague Violence Scale, EVPM = effects of violence on physical and mental health dimension, NTSS = Nursing Transition Shock Scale, PIIR = perception of inadequacy in interpersonal relationships dimension, PIPD = perception of inadequacy in professional decisions and practices dimension, PIRR = perception of inadequacy in roles and responsibilities dimension, PISL = perception of inadequacy in social life dimension, VVD = verbal/psychological violence dimension.

⁣^∗^*p* < 0.05.

**Table 4:** Estimates of the proposed model.

Structural relationship			Standard estimate	Unstandardized estimate	S.E.	C.R.	**p**
Exposure to colleague violence	<---	Perception of inadequacy in interpersonal relationships	0.622	1966	0.217	9.074	⁣^∗∗∗^
Exposure to colleague violence	<---	Perception of inadequacy in professional decisions and practices	−0.12	−0.691	0.314	−2.202	0.028
Exposure to colleague violence	<---	Perception of inadequacy in social life	0.287	1409	0.283	4.982	⁣^∗∗∗^
Exposure to colleague violence	<---	Perception of inadequacy in roles and responsibilities	0.078	0.537	0.415	1.295	0.195

*Note:* The symbols ^∗∗∗^ in Table 4 represent the level of statistical significance for the relationships presented. Specifically, a single asterisk (^∗^) indicates a *p* value less than 0.05, denoting statistical significance. Double asterisks (^∗∗^) represent a *p* value less than 0.01, and triple asterisks (^∗∗∗^) denote a *p* value less than 0.001, indicating a high level of statistical significance. These notations are used to highlight the strength of the associations between the variables.

## Data Availability

The data that support the findings of this study are not publicly available due to privacy and ethical restrictions. The data were collected under the assurance that participants' information would remain confidential and would not be shared. However, data may be made available by the corresponding author upon reasonable request and with permission from the ethics committee.
